# Uptake and metabolism of ciclesonide and retention of desisobutyryl-ciclesonide for up to 24 hours in rabbit nasal mucosa

**DOI:** 10.1186/1471-2210-7-7

**Published:** 2007-06-06

**Authors:** Hideyuki Sato, Ruediger Nave, Takashi Nonaka, Nishibe Yoshihisa, Nagano Atsuhiro, Tsutomu Mochizuki, Shigehiro Takahama, Shiro Kondo, Mark Wingertzahn

**Affiliations:** 1 Teijin Institute for Biomedical Research, TEIJIN Pharma Limited, 4-3-2 Asahigaoka, Hino, Tokyo 191-8512, Japan; 2 ALTANA Pharma AG, Byk-Gulden-Str. 2, 78467 Konstanz, Germany; 3 ALTANA Pharma, 220 Park Avenue, Florham Park, NJ 07932, USA

## Abstract

**Background:**

The nasal tissue uptake and metabolism of ciclesonide, a new-generation corticosteroid under investigation for treatment of allergic rhinitis, to its active metabolite, desisobutyryl-ciclesonide (des-CIC), was evaluated when administered to rabbits in a hypotonic versus an isotonic ciclesonide suspension. Nasal mucosa extracts from normal Japanese white rabbits were evaluated by high-performance liquid chromatography with tandem mass spectrometry detection after a single 143-μg dose of ciclesonide. Retention and formation of fatty acid conjugates of des-CIC were also measured in nasal mucosa extracts postadministration of a hypotonic ciclesonide suspension (143-μg single dose).

**Results:**

Versus an isotonic suspension, the hypotonic suspension achieved higher concentrations of des-CIC (5.6-fold, 11.4-fold, and 13.4-fold; *p *< 0.05 for all) and ciclesonide (25.3-fold, 34.2-fold [*p *= not significant], and 16-fold [*p *< 0.05]) at 30, 120, and 240 min postadministration. Additionally, when administered via a hypotonic suspension, des-CIC was retained up to 24 h postadministration (45.46 pmol/g tissue). Highest concentration of major fatty acid ester conjugate, des-CIC-oleate, was detected in nasal mucosa at 8 h postadministration.

**Conclusion:**

These data suggest that a hypotonic ciclesonide suspension provides higher intracellular concentrations of des-CIC up to 24 h, thereby providing a rationale for investigation of ciclesonide as a convenient once-daily nasal spray for treatment of allergic rhinitis.

## Background

Allergic rhinitis (AR) affects 10% to 30% of the world's population and an estimated 20 to 40 million people in the United States alone ([1-3]. Common symptoms of AR include sneezing, itching, rhinorrhea, and nasal congestion. According to the Allergic Rhinitis and Its Impact on Asthma guidelines, AR is a major chronic respiratory disease that is increasing in prevalence; reduces quality of life, performance, and productivity; and is a significant risk factor for asthma [4,5]. Current pharmacologic treatments for AR include antihistamines, decongestants, and intranasal corticosteroids (INS)[6]. Intranasal corticosteroids are recommended first-line medications in patients with moderate or severe AR and provide effective relief of nasal symptoms and congestion because of their potent anti-inflammatory activity [6-8].

**Figure 1:**
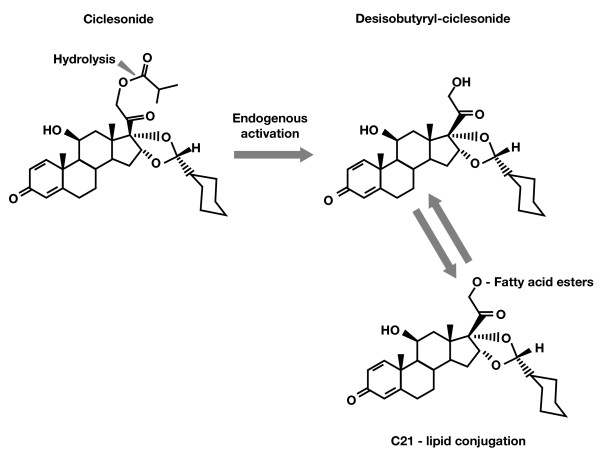
Bioactivation of ciclesonide to the active metabolite, desisobutyryl-ciclesonide (des-CIC), and formation of fatty acid esters of des-CIC.

Ciclesonide is a new-generation corticosteroid currently used for the treatment of asthma and has been approved for the treatment of AR [9-11]. Ciclesonide is administered as an inactive parent compound and is activated by esterases to a pharmacologically active metabolite, desisobutyryl-ciclesonide (des-CIC; Fig. 1) [12]. Esterification of des-CIC at the carbon-21 position produces reversible lipid conjugates of des-CIC that represent a pool of active drug available for sustained long-term anti-inflammatory activity [13]. Studies with ciclesonide in normal human bronchial epithelial cells and rat lung tissue and budesonide formulations used for treatment of AR in human nasal tissue have confirmed the role of fatty acid conjugates in prolonged retention of these drugs [13-15].

The efficacy, safety, and tolerability of INS are affected in part by the formulation of the suspension. Intranasal corticosteroids are usually formulated and administered via an isotonic medium [16-18] However, ciclesonide is formulated in a hypotonic suspension. The viscous consistency of the hypotonic suspension provides longer retardation of ciclesonide on nasal mucosa and a high local concentration of ciclesonide to increase its absorption into nasal mucosa [19]. This may lead to enhanced uptake of ciclesonide in nasal mucosa and higher local tissue concentrations of des-CIC compared with traditional isotonic INS suspensions, theoretically decreasing systemic availability.

Formulation differences exist between INS based on the type of preservative used [20,22]. Unlike other INS that usually contain benzalkonium chloride as the preferred preservative agent [20,21], ciclesonide hypotonic suspension is formulated for intranasal use with potassium sorbate as the preservative agent [22].

The major aim of these preclinical studies in rabbits was to compare the metabolism of ciclesonide to its active metabolite, des-CIC, after intranasal administration as a hypotonic or isotonic suspension and to investigate the retention of ciclesonide and des-CIC and nasal mucosal formation of fatty acid conjugates of des-CIC up to 24 h after intranasal administration of a hypotonic suspension of ciclesonide. Ciclesonide and des-CIC were analyzed in homogenates prepared from the nasal mucosal tissue using liquid chromatography with tandem mass spectrometry. A comparative analysis of data obtained from rabbit nasal mucosa after intranasal administration of hypotonic or isotonic suspension is reported herein.

## Results

### Ciclesonide uptake and formation of des-CIC in rabbit nasal mucosa

**Figure 2:**
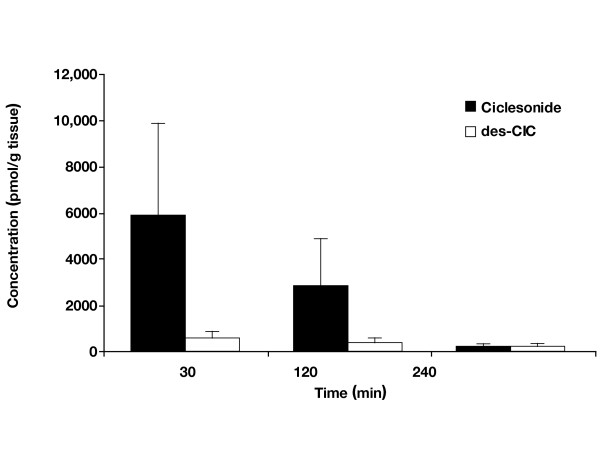
Concentrations of ciclesonide and desisobutyryl-ciclesonide (des-CIC) in rabbit nasal mucosa after single-dose administration of a hypotonic ciclesonide suspension. Bars represent mean concentration ± standard deviation.

Mean nasal mucosal concentration of des-CIC was significantly higher after administration of the hypotonic suspension compared with the isotonic suspension at all time points 5.6-fold, 11.4-fold, and 13.4-fold higher at 30, 120, and 240 min, respectively, after administration; *p *< 0.05 for all; Table 1; Fig. 2). Ciclesonide levels were measurable in all the nasal mucosa samples only at 30 min after administration of isotonic suspension. However, 1 and 2 of the 4 samples were below the lower limit of quantification at 120 and 240 min, respectively. By contrast, when administered in hypotonic suspension, ciclesonide was measurable in the nasal mucosa at all time points (Table 2). The concentration of ciclesonide was 25.3-fold higher at 30 min and 34.2-fold higher at 120 min after administration of the hypotonic suspension compared with the isotonic suspension (*p *= not significant). Moreover, the concentration of ciclesonide was significantly higher at 240 min (16-fold) after administration of the hypotonic suspension compared with the isotonic suspension (*p *< 0.05).

**Table 1:** Administration of hypotonic and isotonic ciclesonide suspensions and nasal tissue sampling.

Suspension administered	Animals, n *	Dose per animal, μg	Volume per actuation, μL	Sampling time, h
Hypotonic ciclesonide	4	143	100	0.5, 2, 4
Isotonic ciclesonide	4	143	100	0.5, 2, 4
Hypotonic ciclesonide	5	143	100	0.5, 8, 12, 16, 24

* = Represents the number of animals per sampling time.

**Table 2:** Concentrations of ciclesonide and des-CIC in rabbit nasal mucosa.

Time, min	Ciclesonide pmol/g tissue *	*p *value **	des-CIC pmol/g tissue *	*p *value **
Hypotonic suspension				
30	5884 ± 3982	NS	575 ± 276.3	< 0.05
120	2835 ± 2118	NS	366 ± 207	< 0.05
240	212.5 ± 102.1	< 0.05	215.2 ± 112.2	< 0.05
Isotonic suspension				
30	232.3 ± 46.3	-	102 ± 32.1	-
120	83 ± 69.1	-	32 ± 25	-
240	13.3 ± 16.4	-	16 ± 20.3	-

des-CIC = desisobutyryl-ciclesonide; NS = not significant.

* = Data are mean ± standard deviation.

** = Versus isotonic suspension.

### Concentration-time profile of des-CIC in nasal mucosa

The concentration-time profiles of des-CIC in nasal mucosa in the hypotonic and isotonic suspensions were distinctly different. For the hypotonic ciclesonide suspension, a relatively high amount of des-CIC (215.2 pmol/g tissue, 2.7-fold lower) was detected in the nasal mucosa 240 min after administration compared with 30 min (575 pmol/g tissue) after administration (Table 2). In contrast, when administered in isotonic suspension, a negligible amount of des-CIC (16 pmol/g tissue, 6.5-fold lower) was detected in the nasal mucosa 240 min after administration compared with 30 min (102 pmol/g tissue) after administration.

### Retention of des-CIC and des-CIC fatty acid conjugates in rabbit nasal mucosa

**Figure 3:**
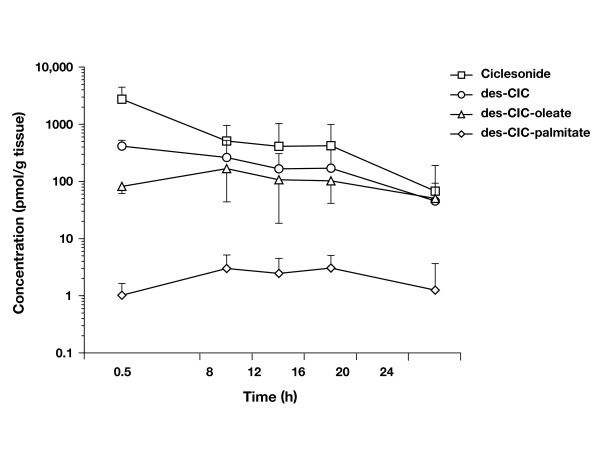
Concentrations of ciclesonide, desisobutyryl-ciclesonide (des-CIC), and fatty acid conjugates of des-CIC in rabbit nasal mucosa after single-dose administration of a hypotonic ciclesonide suspension. Values represent mean concentration ± standard deviation.

Desisobutyryl-ciclesonide was measurable in the nasal mucosa at all time points up to 24 h (45.46 pmol/g tissue) after administration of a hypotonic suspension of ciclesonide (Fig. 3). The highest concentration of des-CIC (415 pmol/g tissue) was detected in the nasal mucosa as early as 0.5 h after administration of ciclesonide, suggesting that ciclesonide is rapidly converted to the active metabolite. Desisobutyryl-ciclesonide-oleate and des-CIC-palmitate were measurable in the nasal mucosa at all time points up to 24 h after administration of ciclesonide. Desisobutyryl-ciclesonide-oleate was the major fatty acid conjugate formed in the nasal mucosa, and the highest concentrations of des-CIC-oleate (167 pmol/g tissue) and des-CIC-palmitate (3.05 pmol/g tissue) were detected in the nasal mucosa 8 h and 16 h, respectively, after administration of a hypotonic ciclesonide suspension.

## Discussion

The intracellular localization and sustained concentration of INS are dependent to varying levels on nasal mucosal absorption, drug lipophilicity, glucocorticoid receptor affinity, and the ability to form lipid conjugates in nasal epithelial cells [23]. Retention and formation of fatty acid conjugates of INS in human nasal tissue and its importance in treatment of AR have been reported [15,24]. In the present study, the *in vivo *uptake and activation of ciclesonide in a hypotonic suspension have been shown to be superior to ciclesonide's uptake and activation in an isotonic suspension. Furthermore, it has also been shown that des-CIC and fatty acid conjugates of des-CIC are present in rabbit nasal mucosa up to 24 h after administration of ciclesonide in a hypotonic suspension.

Nasal mucosal concentrations of ciclesonide were numerically higher after administration of a hypotonic suspension compared with an isotonic suspension and decreased after 240 min of administration. The presence of relatively higher amounts of ciclesonide versus des-CIC 120 min after administration of the hypotonic suspension may be explained by better absorption of ciclesonide in hypotonic medium. The presence of relatively smaller amounts of ciclesonide (232.5 pmol/g tissue) at 30 min after administration of the isotonic suspension suggests slow uptake of ciclesonide when administered in isotonic suspension. However, the nasal mucosal concentrations of des-CIC were significantly and consistently higher 240 min after administration of a hypotonic suspension. There is limited data available on the influence of tonicity on nasal uptake [25,26]and tonicity is unlikely to have any effect on particle size distribution. However, previous studies in other systems have demonstrated that hypotonic formulations promote significantly greater absorption of water and sodium in the intestine [27,28].

The presence of des-CIC in nasal mucosa as early as 30 min after administration of hypotonic suspension suggests rapid conversion of ciclesonide. The identification of esterases present in nasal tissue is currently being investigated in human nasal epithelial cells; although intracellular esterases are highly conserved across the species, it would not be surprising to see minor differences in human nasal mucosa. Preliminary evidence suggests that carboxylesterases and cholinesterases are involved in the metabolism of ciclesonide to des-CIC in human nasal epithelial cells [29].

Our subsequent experiment with hypotonic suspension in rabbit nasal mucosa further corroborated the evidence of metabolism of ciclesonide and confirmed that des-CIC is retained in nasal mucosa for 24 h after a single-dose administration. This study also confirmed the formation of des-CIC-oleate and des-CIC-palmitate conjugates of des-CIC in nasal mucosa, reaching their highest levels 8 h and 16 h postadministration, respectively. The presence of fatty acid conjugates of des-CIC suggests that the active drug may be retained in the nasal mucosa as a reversible pool to exert sustained drug effects over time and allow once-daily dosing. Similar metabolism of ciclesonide to des-CIC in normal human bronchial epithelial cells and rat lung tissue [14] and the presence of des-CIC-oleate and des-CIC-palmitate fatty acid conjugates of des-CIC for a longer period of time have also been demonstrated in rat lung tissue [13,30]. Fatty acid conjugation has also been reported for budesonide in lung and nasal tissue [24,31]. However, des-CIC-oleate is 5-fold more lipophilic than budesonide-oleate [32].

**Figure 4:**
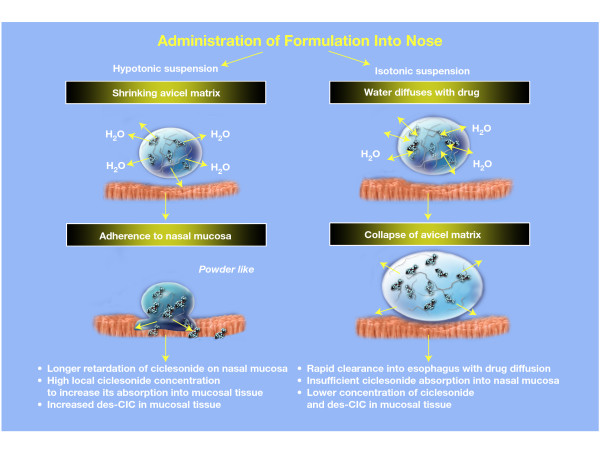
The hypothetical mechanism of the intranasal uptake of hypotonic or isotonic suspensions of ciclesonide. des-CIC, desisobutyryl-ciclesonide.

The hypotonic ciclesonide suspension containing the avicel matrix used in this study diffuses rapidly into nasal mucosa because of a difference in osmolarity between the suspension and the nasal mucosa [19]. Diffusion of water and subsequent loss of water into the nasal mucosa lead to increased viscosity of the suspending agent and higher adherence to nasal mucosa. This higher adherence of the suspending agent leads to rapid accumulation of ciclesonide in the nasal mucosa and probable delayed clearance of ciclesonide (Fig. 4). We hypothesize that initial conversion of ciclesonide to des-CIC occurs almost instantaneously, whereas the subsequent conversion of des-CIC to des-CIC-oleate and des-CIC-palmitate occurs slowly. Theoretically, the interconversion of des-CIC from the fatty acid conjugates may lead to relatively higher concentrations of des-CIC within nasal mucosa for a longer period of time.

## Conclusions

The results of this study confirm that a hypotonic ciclesonide suspension provides better absorption and results in higher cellular levels of ciclesonide and its active metabolite, des-CIC, in nasal mucosa compared with an isotonic ciclesonide suspension. The presence of des-CIC and fatty acid conjugates of des-CIC in the rabbit nasal mucosa up to 24 h after a single administration provides a rationale for the investigation of ciclesonide nasal spray administered via a convenient once-daily dosing regimen for the treatment of AR.

## Methods

This is a combined presentation of 2 related studies. The first was a comparison of hypotonic and isotonic suspensions of ciclesonide and will be referred to as the "comparison" study. The second was a further evaluation of the ability of des-CIC to form fatty acid conjugates in nasal mucosa when ciclesonide is administered in hypotonic suspension. The duration of fatty acids and des-CIC retention was also evaluated, and herein this study is referred to as the "retention" study.

### Materials

Ciclesonide, des-CIC, deuterated des-CIC, des-CIC-oleate, and des-CIC-palmitate were supplied by ALTANA Pharma AG (formerly Byk Gulden, Konstanz, Germany). Hypotonic and isotonic ciclesonide suspensions containing the avicel matrix were supplied by Teijin Institute for Biomedical Research, Teijin Pharma Limited, Tokyo, Japan (formerly Teijin Limited, Tokyo, Japan). Differences between the 2 formulations were based on osmolarity of glucose and the viscosity of microcrystalline cellulose (Table 3).

**Table 3:** Composition of hypotonic and isotonic formulations of ciclesonide.

Composition, % weight	Hypotonic	Isotonic
Crystalline cellulose/Carmellose sodium	1.7	1.7
Ciclesonide	0.0714	0.0714
HPMC	0.1	0.1
Glucose	4.75	-
pH	4.53	4.54
Viscosity (mPa/s)	94	76
Osmolality (mOsm)	324	33

HPMC = Hydroxypropyl methylcellulose.

### Animals

Male (specific pathogen free) Japanese white rabbits, 16 to 17 weeks old, 2.5 to 3.1 kg weight were supplied by Japan SLC Inc. (Shizuoka, Japan). Animals were individually housed in cages (AM-2612-03, Animec, Tokyo, Japan), and water was provided *ad libitum*. Solid feed (LRC4, Oriental Yeast Co., Ltd., Tokyo, Japan, for the retention study and CR-3, Japan CLEA Inc., Tokyo, Japan, for the comparison study) for rabbits was provided *ad libitum*. The temperature in the room was 24 ± 2°C, and the relative humidity was 55% ± 5% for the comparison study. The temperature in the room was 24 ± 2°C, and the relative humidity was 55% ± 15% for the retention study. The animal-room lighting followed a 12-h light/dark cycle. Animals were 17 weeks old at the time of administration of hypotonic or isotonic ciclesonide suspensions or were 19 weeks old at the time of the retention study. The guidelines established by the ethical committee for animal experiments of the Teijin Institute for Biomedical Research were followed for care and use of animals.

### Study design

All studies were performed at the Teijin Institute for Biomedical Research. Hypotonic and isotonic ciclesonide suspensions were administered intranasally using a nasal actuator (Flunase^® ^Nasal Solution50, approved in Japan; GlaxoSmithKline K.K., Tokyo, Japan; Table 3). One-hundred microliters of the suspension was applied to each nostril to obtain consistent saturation of the nasal mucosa.

### Tissue sampling

In the comparison study, at prescribed time points after intranasal administration of hypotonic and isotonic ciclesonide suspensions (Table 1), nostrils were washed 3 times with 5 mL of physiological saline. Animals were anesthetized with pentobarbital sodium and exsanguinated from the carotid artery. Nasal mucosa including ventral nasal concha, middle nasal meatus, choana, and olfactory mucosal were immediately removed, weighed, suspended in 4-fold volume of ice-cold ethanol of the wet weight, minced, and homogenized. One milliliter of the resultant homogenate was mixed with 25 μL of deuterium-labeled des-CIC internal standard solution (2 μg/mL) in ethanol, vortex-mixed, and centrifuged at 1660 × *g *for 15 min at 4°C. The extracted supernatant was stored at -20°C until further analysis.

In the retention study, at prescribed time points after intranasal administration of ciclesonide in a hypotonic suspension (Table 3), animals were anesthetized with 33% (w/v) urethane aqueous solution containing 0.83% (w/v) α-chloralose and were exsanguinated from the abdominal aorta. Immediately thereafter, the inside of the nasal cavity was washed 5 times with 30 mL of physiologic saline containing 600 μmol/L phenylmethyl sulfonyl fluoride (a serine protease inhibitor) using a cannula. Nasal mucosa, including the nasal septum; ventral, middle, and dorsal nasal meatus and concha; and internal nasal concha was immediately removed. Wet weight of nasal mucosa was determined and was frozen in liquid nitrogen and stored at -20°C until further analysis. For extraction, nasal mucosa was minced in 4-fold volume of ice-cold ethanol and homogenized. The homogenate was centrifuged at 2620 × *g *for 15 min at 4°C, and 0.5 mL aliquots of the supernatant were dispensed in Eppendorf tubes and stored at -20°C. Seventy-five microliters of each nasal tissue extract solution was combined with 25 μL of deuterated des-CIC internal standard solution (100 ng/mL), and the resultant mixture was used for analyses.

### Analysis by liquid chromatography with tandem mass spectrometry (LC/MS/MS)

For the comparison study, 100 μL of purified water was added to 100 μL of the extracted supernatant and vortex-mixed. The samples were then transferred to 0.45 μm membrane filters (Ultrafree-MC, Millipore Corporation, Billerica, Massachusetts) and were centrifuged at 840 × *g *for 3 min. The filtered samples were then transferred to injection vials and measured by LC/MS/MS. Ciclesonide and des-CIC were separated by high-performance liquid chromatography (Agilent HP1100, Agilent Technologies, Tokyo, Japan) using a CAPCELL PAK UG120, C18 (3 μm, 2 × 50 mm ID) column (Shiseido Company Limited, Tokyo, Japan) with a mobile phase consisting of 65% (v/v) acetonitrile/ethanol (4:1 ratio) and 35% (v/v) 5 mmol/L ammonium acetate solution at a flow rate of 250 μL/min. Calibration standards contained ciclesonide, des-CIC, and deuterated des-CIC (analytes: ciclesonide and des-CIC; internal standard: deuterated des-CIC, range 0.5 ng/mL to 1 μg/mL). The lower limits of quantification were 1.0 and 0.5 ng/mL for ciclesonide and des-CIC, respectively. Ciclesonide and des-CIC were detected by turbo ion spray MS/MS (API3000, Applied Biosystems, Tokyo, Japan) with negative polarity and multiple reaction monitoring scan mode.

For the retention study, concentrations of ciclesonide, des-CIC, and fatty acid conjugates (des-CIC-oleate and des-CIC-palmitate) in the supernatants from nasal mucosal homogenates were analyzed by LC/MS/MS. Ciclesonide, des-CIC, and des-CIC fatty acid conjugates were separated by high-performance liquid chromatography (Agilent, HP1100) using a Hypersil^® ^Phenyl2 (5 μm, 4.6 × 50 mm ID) column (Thermo Electron, K. K., Yokohama, Japan) with a mobile phase consisting of 95% (v/v) acetonitrile/1 mmol/L ammonium acetate solution and 25% (v/v) acetonitrile/1 mmol/L ammonium acetate solution at a flow rate of 1 mL/min for a total run time of 7 min. Calibration standards contained ciclesonide, des-CIC, des-CIC-oleate, des-CIC-palmitate, and deuterated des-CIC (analytes: ciclesonide, des-CIC-oleate, and des-CIC-palmitate; internal standard: deuterated des-CIC, range 0.1 to 20 ng/mL, and des-CIC, range 0.2 to 40 ng/mL). The lower limits of quantification were 0.1 ng/mL for ciclesonide, 0.2 ng/mL for des-CIC, and 0.10 ng/mL for both des-CIC-oleate and des-CIC-palmitate, respectively. Ciclesonide and its metabolites were detected by turbo ion spray MS/MS (API3000, Applied Biosystems, Tokyo, Japan) with negative polarity and multiple reaction monitoring scan mode. Molecular weights of ciclesonide, des-CIC, des-CIC-oleate, and des-CIC-palmitate are 540.7, 470.6, 735.1, and 709.0 g/mol, respectively.

### Data analysis

Calibration curves were prepared by the linear least squares method using the peak area ratios of the internal standard solutions. Concentrations of analytes in the samples were calculated by substituting the peak area ratios determined by a quantitative analysis versus the calibration curve. Data were calculated as the concentration of analytes versus unit nasal mucosal weight after converting concentration (ng/mL) to corresponding molar concentrations and expressed as mean ± standard deviation. Unpaired *t *tests for mean value of nasal mucosal concentration in both hypotonic and isotonic suspension in the comparison study were performed using the SAS (version 6.12) software program (SAS Institute Inc, Cary, North Carolina).

## Authors' contributions

HS, TN, NY, NA, TM, ST, and SK participated in the study design, carried out animal experiments, analyzed tissues by liquid chromatography with tandem mass spectrometry, performed statistical analysis, and participated in drafting the manuscript. RN and MW participated in data analysis and drafting of the manuscript. All authors have read and approved the final manuscript.
